# Discovery of a novel Keap1 inhibitor for neurodegeneration through virtual screening and molecular dynamics simulations

**DOI:** 10.1371/journal.pone.0341965

**Published:** 2026-02-02

**Authors:** Md. Mazedul Hasan, Md. Shaki Mostaid, Asim Kumar Bepari, Hasan Mahmud Reza, Murad Hossain

**Affiliations:** Department of Pharmaceutical Sciences, North South University, Dhaka, Bangladesh; George Washington University, UNITED STATES OF AMERICA

## Abstract

Oxidative stress is a key feature of Alzheimer’s disease (AD) and other neurodegenerative disorders. The Kelch-like ECH-associated protein 1 (Keap1)–nuclear factor erythroid 2–related factor 2 (Nrf2) pathway controls redox balance, and disrupting the Keap1–Nrf2 protein–protein interaction (PPI) has become a promising therapeutic approach. Marine natural products (MNPs), because of their structural diversity and bioactivity, are an underexplored source of potential neuroprotective compounds. This study aimed to identify novel marine-derived inhibitors of the Keap1–Nrf2 interaction using a comprehensive in silico pipeline. A total of 14,492 compounds from an open-access MNP database were virtually screened against the Keap1 Kelch domain through molecular docking. The top 1,329 candidates exhibited strong binding affinities, with several reaching scores comparable to the co-crystallized reference ligand L5F. Absorption, distribution, metabolism, excretion, and toxicity (ADMET) profiling was employed to assess pharmacokinetic properties, brain penetration, and safety, leading to the identification of compound 145398-61-4 as the most promising hit. Molecular dynamics (MD) simulations verified the structural stability of the Keap1–145398-61-4 complex, while binding free energy calculations indicated energetically favorable interactions. Additional validation using principal component analysis (PCA) and highest occupied molecular orbital–lowest unoccupied molecular orbital (HOMO–LUMO) energy analysis further confirmed the stability of this interaction. Overall, our in silico study identified compound 145398-61-4 as a novel Keap1–Nrf2 inhibitor, highlighting its potential as a lead candidate for developing treatments for Alzheimer’s disease and other neurodegenerative disorders, such as amyotrophic lateral sclerosis and multiple sclerosis.

## Introduction

With an estimated 50 million cases worldwide, Alzheimer’s disease (AD) is the most common form of dementia and the sixth leading cause of death in the Western world [1], representing a major public health issue worldwide [2]. Factors contributing to the pathophysiology of neuronal malfunction and degeneration associated with AD include oxidative damage, endoplasmic reticulum (ER) stress, and mitochondrial dysfunction [3–5]. AD patients with mild cognitive impairment (MCI) also show signs of oxidative stress in their brains, plasma, and erythrocytes [6,7]. Elevated oxidative stress markers have been reported in many animal studies, including in the pre-plaque deposition brains of transgenic AD mice [8,9]. It is believed that oxidative damage occurs during the early stages of AD and that the disease may present as a redox imbalance [10]. Both humans and experimental animals with AD have demonstrated reduced cellular antioxidant capacity and increased production of free radicals in their brains [11,12]. As a result, targeting key regulators of cellular oxidative stress may provide a promising approach for improving therapeutic interventions for neurodegenerative disorders [13].

Kelch-like ECH-associated protein 1 (Keap1) and nuclear factor erythroid 2-related factor 2 (Nrf2) are two key regulators of oxidative stress [14,15], recognizing Nrf2 as a therapeutic target in conditions associated with redox imbalance [16]. Under physiological conditions, Nrf2 binds to Keap1, rendering it inactive and leading to its degradation. When Nrf2 detaches from the Keap1-Nrf2 complex in response to electrophile or free radical stress, it moves into the nucleus and binds to the antioxidant response element (ARE), modulating the transcription and production of antioxidants and phase II detoxifying enzymes [17]. As a result, abnormal Nrf2 signaling plays a crucial role in the pathophysiology of AD by contributing to the intolerance of oxidative stress [18–20]. A study conducted in 2007 observed decreased Nrf2 expression in the hippocampal area of transgenic APP/PS1 (amyloid precursor protein/presenilin 1) mice [21]. Introducing lentiviral-vectored Nrf2 into the hippocampal regions of AD mice improves their spatial learning [22]. Additionally, the brain tissue of AD patients shows significantly lower levels of nuclear Nrf2, as well as reduced Nrf2 mRNA and protein levels, compared to normal brain tissue [23].

Existing evidence strongly suggests that in neurodegenerative diseases like AD, the protein-protein interaction (PPI) between Keap1 and Nrf2 is a crucial therapeutic target [2,13,16]. We used standard computational methods to find Keap1 inhibitors in our study. Marine natural products, or MNPs, are chemical compounds derived from sponges, cnidarians, fungi, bacteria, and other aquatic organisms [24]. In modern drug development pipelines, MNPs are considered vital sources for new compounds because of their chemodiversity [25–28]. Approximately 30% of the MNPs analyzed in recent preclinical trials showed medium to high enzyme inhibition, and over a thousand compounds demonstrated anti-inflammatory activity [24]. For example, the gracilins from marine sponges have been shown to inhibit beta-secretase 1 (BACE1), thereby reducing inflammation and oxidative stress in vitro [29]. Another MNP that has garnered significant research is astaxanthin, known to decrease neuroinflammation and enhance cognitive symptoms of AD [30]. At least eight marine products are approved as drugs, with several more undergoing pre-clinical and clinical research at various stages (ClinicalTrial.gov).

Considering the critical role of the Keap1–Nrf2 axis in managing oxidative stress and its link to Alzheimer’s disease, targeting their interaction is a promising therapeutic strategy. MNPs, known for their chemodiversity and significance as sources of bioactive compounds, offer a valuable resource for discovering new inhibitors. In this study, we systematically analyzed an open-access MNP database to find potential inhibitors of the Keap1–Nrf2 interaction. We employed molecular docking, in silico ADMET assessments, and atomistic molecular dynamics simulations to identify marine-derived compounds with favorable pharmacological profiles, aiming to identify lead candidates for the development of new Alzheimer’s treatments. To identify potential Keap1-Nrf2 interaction inhibitors that could aid in the development of drugs for Alzheimer’s and other similar neurodegenerative disorders, we conducted computational screening of an open-access MNP database using molecular docking, ADMET predictions, and atomistic simulations.

## Materials and methods

### Protein 3D structure

We obtained the human Keap1 pdb file 8A46 [31] from the RCSB Protein Data Bank (https://www.rcsb.org/structure/8A46). This 3D protein structure was generated via X-ray diffraction, with a resolution of 1.32 Å, and includes the co-crystallized ligand S217879 (L5F), a potent inhibitor of the Keap1-Nrf2 pathway [31].

### Ligand library

We used the Marine Natural Products (MNP) library (Marvin annotated) in our study, which was collected from a previous study [32] and was initially available on the Molecular Docking website (http://docking.umh.es/downloaddb) of the Molecular and Cell Biology Institute at Miguel Hernández University (UMH), Elche, Spain. The retrieved SDF file contained 14,492 small molecules (S1 File). For the compounds in the MNP library, CAS numbers serve as identifiers. We used Openbabel [33] to exclude entries with molecular weights below 150 or over 1,000 to create an initial database that included 13,314 molecules.

### Molecular docking

Marine ligands were prepared for molecular docking using Open Babel (version 3.1.1) [33]. Hydrogen atoms were added, and the Weighted Rotor Search method was used to generate three-dimensional conformers. We employed MMFF94, a variant of the Merck Molecular Force Field (MMFF), for parameterization. Ligands were then energy-minimized using 5,000 steps of the steepest descent algorithm. The protein structures were processed using AutoDockTools (version 1.5.7) to eliminate water molecules, bound ligands, and unwanted chains. After assigning Kollman United Atom charges, the protein was saved in the pdbqt format.

The ligands were then subjected to AutoDock Vina (version 1.5.7) molecular docking [34]. The ligand L5F served as the reference standard. A 28 Å x 28 Å x 28 Å grid box was centered at the XYZ coordinates −1.94, 47.833, and −9.08217, respectively, to match the ligand’s position in the crystal structure. Molecular docking using AutoDock Vina was performed with an exhaustiveness of eight. The Discovery Studio Visualizer (BIOVIA Discovery Studio Visualizer v21.1.0.20298, Dassault Systèmes, San Diego, CA, USA) was used to display the protein-ligand interactions.

### Absorption, distribution, metabolism, excretion, and toxicity prediction

For Absorption, distribution, metabolism, excretion, and toxicity (ADMET) profiling of ligands, we used SwissADME [35], Protox-3.0 [35], and the Osiris property explorer [36].

### Molecular dynamics simulation

CHARMM-GUI’s “Solution Builder” module was used [37] for input generation for Gromacs (version 2023.2) molecular dynamics (MD) simulations [38]. We implemented a previously documented MD simulation procedure [39].

The V-rescale thermostat was used after the LINCS constraint algorithm to equilibrate the system for 100 ps at 300 K. The next step was an NPT ensemble utilizing the V-rescale thermostat and C-rescale barostat for 100 ps at 1 bar and 300 K. After the final removal of the constraints, MD simulations were carried out using the same NPT ensemble [40].

We analyzed the MD simulation trajectories focusing on root-mean-square deviation (RMSD), radius of gyration (Rg), hydrogen bonds, root-mean-square fluctuation (RMSF), and principal component analysis (PCA).

### Molecular mechanics-Poisson Boltzmann surface area free energy calculation

We calculated the molecular mechanics-Poisson Boltzmann surface area (MM-PBSA) free energy using the gmx_MMPBSA tool with Gromacs. We sampled 500 frames from the last 10 ns of the trajectory. The gas-phase interaction energy (GGAS), which includes van der Waals and electrostatic energies, along with solvation energy (GSOLV), contributed to the total binding free energy [41,42].

### Molecular orbital energy calculation

We calculated molecular orbital energy following previously reported protocols [43–46] where we calculated the Highest Occupied Molecular Orbital (HOMO) and the Lowest Unoccupied Molecular Orbital (LUMO) energies as well as their energy gaps (ΔE). The initial molecular structures of the Polycyclic Aromatic Hydrocarbons (PAHs) were generated from their SMILES strings using the RDKIT cheminformatics toolkit. Molecular geometry was subsequently optimized using the MMFF94 molecular mechanics force field, after explicitly adding hydrogen atoms to obtain a reasonable starting structure for the quantum chemical calculation. This methodology was implemented using the Python-based Simulations of Chemistry Framework (PySCF) software package. Subsequent single-point energy calculations were conducted at the Restricted Hartree-Fock (RHF) level theory with the STO_3G basis set. This approach solves the self-consistent Hartree-Fock-Roothaan equations, treating each electron in the mean field of the others, to produce molecular orbitals and their energies. The HOMO and LUMO energies were identified directly from the resulting eigenvalues, and the HOMO-LUMO gap was calculated as ΔE = E_LUMO – E_HOMO. The energy gap was converted from the atomic units (Hartree) to electronvolts (eV) using the standard conversion factor of 1 Ha = 27.2114 eV. As we did not conduct any laboratory experiments on humans or animals, ethical approval was not required.

### Statistical analysis

We used one-way ANOVA to compare the means for RMSD, RMSF, and Rg as these are continuous variables analyzed across a single categorical factor.

## Results

### Virtual screening of Keap1-Nrf2 inhibitors

Before docking the candidate ligands from the MNP library, we redocked the reference ligand L5F to Keap1. The docking results showed that our AutoDock Vina protocol could accurately reproduce the binding conformation of the inhibitor, as seen in the crystal structure of Keap1 (PDB ID: 8A46). A projected binding affinity of −13.3 kcal/mol was identified for the reference ligand.

Subsequently, we ranked the ligands based on their binding scores after conducting Vina molecular docking on the MNP library. The binding scores of the 13,314 MNPs ranged from −2 at the lowest to −13.7 at the highest (S2 File). Among these, 1329 compounds with binding scores from −10 to −13.7 were selected for SwissADME (http://www.swissadme.ch) prediction, where their physicochemical properties were estimated.

### ADMET prediction for top ligands

Using SwissADME, we evaluated the physicochemical properties of the 1329 ligands and assessed their druglikeness based on Lipinski’s Rule of Five (Ro5) [47]. Out of these, 252 compounds that violated no more than one of Lipinski’s criteria were selected for further analysis. These 252 molecules had molecular weights ranging from 254 to 495, with 0–9 rotatable bonds. The number of hydrogen bond acceptors varied from 1 to 9, while the number of hydrogen bond donors ranged from 0 to 5. All selected compounds had LogP values below five, indicating favorable lipophilicity profiles (S3 File).

We evaluated in silico toxicity using the Protox web server (https://tox.charite.de/protox3/) and the Osiris Property Explorer. Predictions included mutagenic, carcinogenic, irritant, and reproductive effects, as well as toxicity class and LD50 values for the compounds. Compounds with no or mild toxicity, classified as toxicity class 6, were considered safe. Only 15 compounds met these criteria and are listed in the S4 File. Two ligands, 17942-08-4 and 22785-88-2, showed structural similarity to 102976-85-2 and were excluded from further analysis.

**Fig 1:**
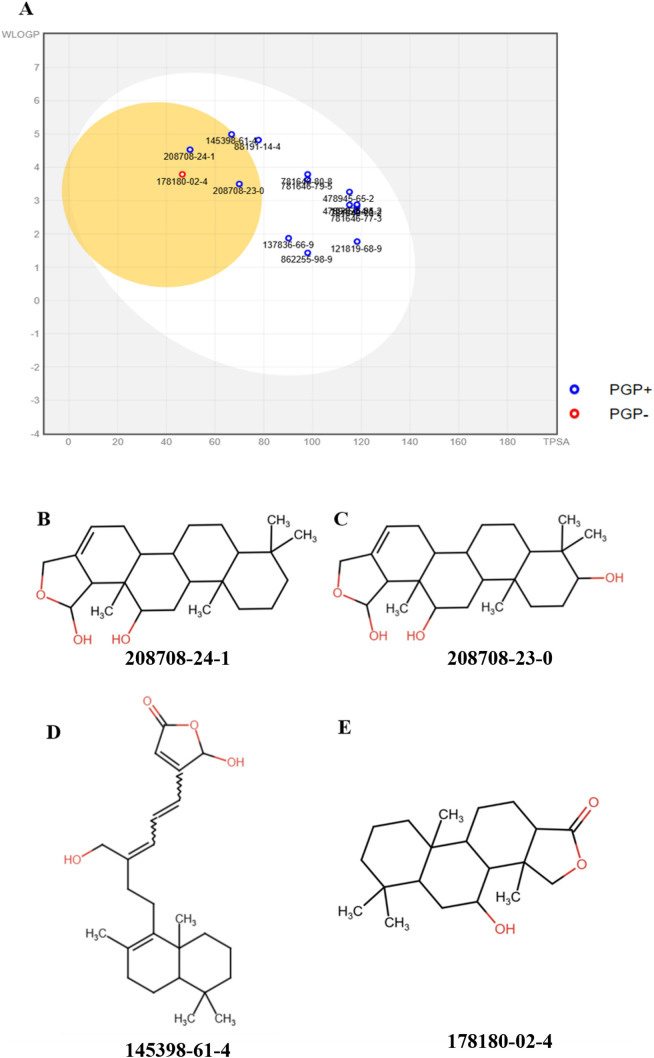
BOILED-Egg analysis and chemical structures of the top-ranked ligands. Top ligands’ TPSA and WLOGP on the BOILED-Egg plot and their chemical structures. (A) The egg yolk represents the permeability of the blood-brain barrier, whereas the egg white signifies oral bioavailability. (B-E) Chemical structures of top ligands: 208708-24-1 (B), 208708-23-0 (C), 145398-61-4 (D), and 178180-02-4 (E).

SwissADME was used to generate a BOILED-Egg plot [48] using the top compounds’ WLOGP values and projected topological polar surface area (TPSA) (Fig 1A). All 15 compounds were located inside the egg, indicating favorable oral bioavailability. Intriguingly, only four compounds, 178180-02-4, 208708-24-1, 208708-23-0, and 145398-61-4, were positioned within the egg yolk region, suggesting their potential to cross the blood-brain barrier (BBB). The chemical structures of these molecules are depicted in Fig 1B-E.

Toxicity prediction showed that the compounds 178180-02-4, 208708-24-1, and 208708-23-0 had no mutagenic, irritating, reproductive toxic, or carcinogenic effects. Conversely, 145398-61-4 exhibited mild carcinogenicity.

### Protein-ligand interaction analysis

**Fig 2:**
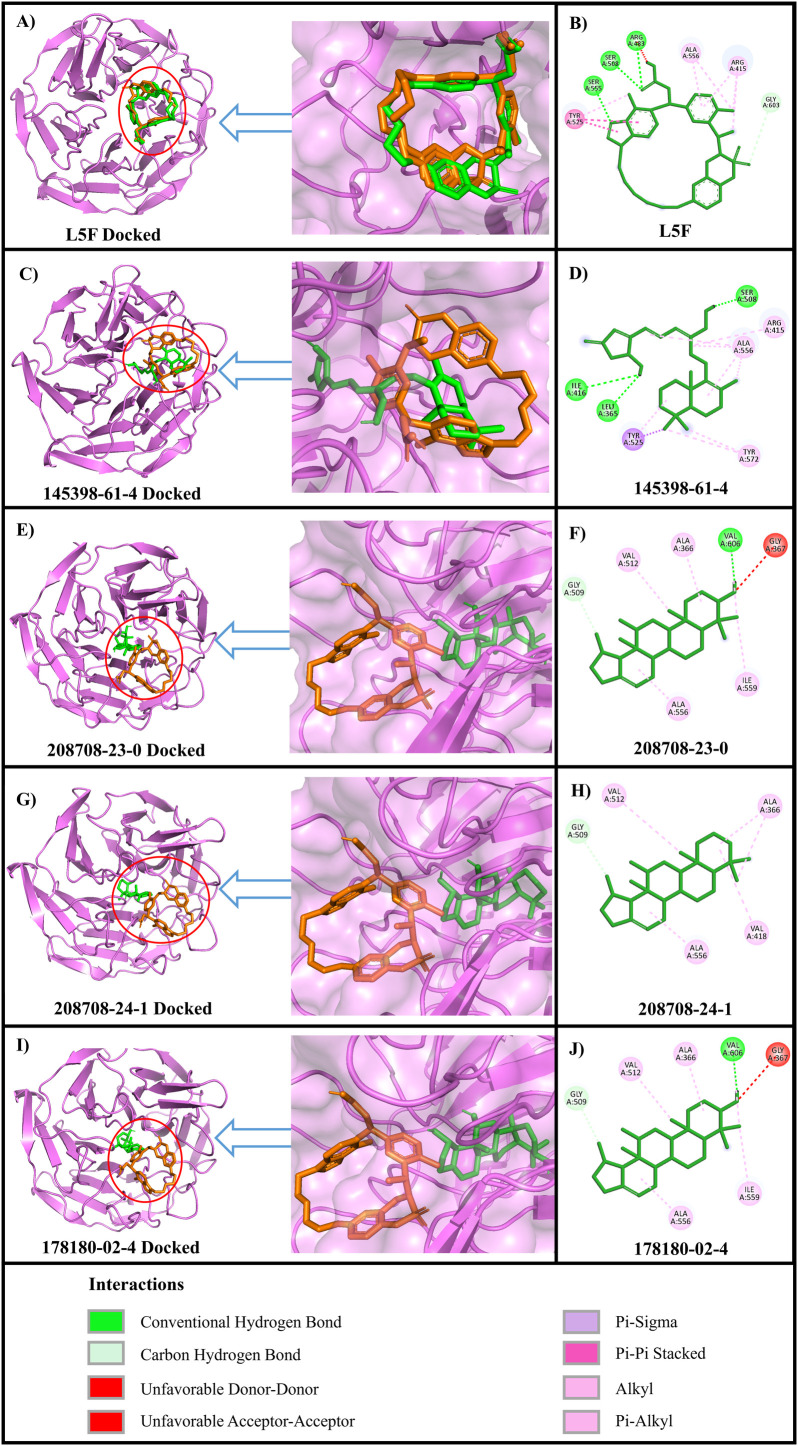
Protein-ligand interactions of the shortlisted ligands. (A, C, E, G, and I) 3D superimposition of L5F in the crystal structure (orange) and docked ligands (green). (B, D, F, H, and J) 2D representations of the protein-ligand interactions.

The hydrogen bonds and hydrophobic interactions between Keap1 and the shortlisted compounds, as well as the reference ligand, were analyzed using BIOVIA Discovery Studio Visualizer and are shown in Table 1 and Fig 2. The reference ligand L5F formed eight hydrophobic interactions and five hydrogen bonds with Keap1. Among the four identified hits, compound 145398-61-4 showed the highest similarity to L5F in terms of interactions. Notably, 145398-61-4 formed nine hydrophobic interactions and three hydrogen bonds. Interestingly, both L5F and 145398-61-4 were predicted to form a key hydrogen bond with the SER508 residue of Keap1, located within the polar sub-pocket P1, one of the six binding sub-pockets of Keap1 [49]. These two ligands also form hydrophobic interactions with TYR525, a crucial binding spot within the Keap1-Nrf2 PPI interface [50].

**Table 1:** H-bond and hydrophobic interaction analysis.

Complex	H-bond interactions	Hydrophobic interactions
	Amino acid	Amino acid
8A46 with L5F	ARG483 SER508 SER508 SER555 GLY603	TYR525 TYR525 ALA556 ARG415 ARG415 TYR525 ARG415 ALA556
8A46 with 178180-02-4	VAL606 GLY509	ALA366 ALA556 VAL512 ILE559 VAL606
8A46 with 208708-24-1	GLY509	ALA366 ALA366 VAL418 ALA556 VAL512
8A46 with 208708-23-0	VAL606 GLY509	ALA366 ALA556 VAL512 ILE559 VAL606
8A46 with 145398-61-4	LEU365 ILE416 SER508	TYR525 ARG415 ALA556 ALA556 ALA556 ALA556 TYR525 TYR572 TYR572

### MD simulation

**Fig 3:**
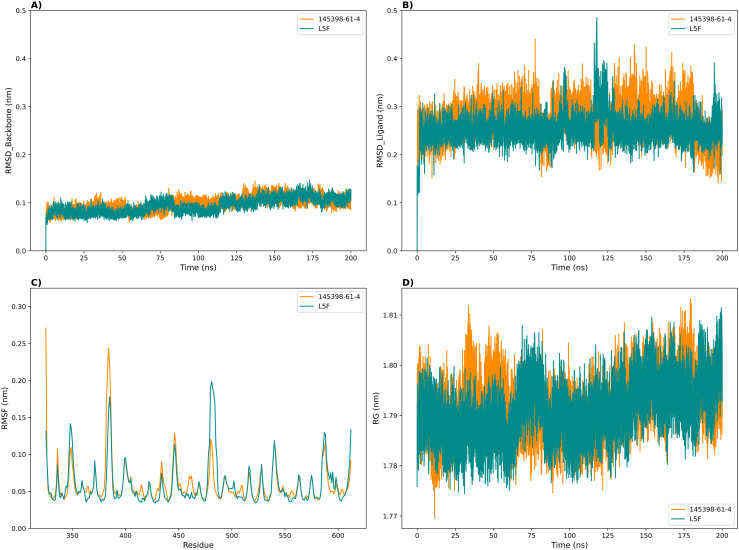
Spatial variations of Keap1 and the ligands during MD simulation of complexes. (A) Protein backbone RMSD. (B) Ligand RMSD (using the protein backbone as the reference). (C) Protein backbone RMSF and (D) Protein radius of gyration (Rg).

We conducted 200 ns all-atom molecular dynamics simulations of the Keap1-L5F crystal structure and the 145398-61-4-Keap1 docked complex using Gromacs and analyzed the trajectories. RMSD calculations provide reliable information on the stability of the molecules and the system’s equilibration. [51–53]. For both complexes, 145398-61-4 and L5F, the protein backbone RMSD values remained consistently low, averaging 0.097 ± 0.014 nm and 0.093 ± 0.015 nm, respectively—indicating high structural stability throughout the simulation (Fig 3A). Ligand RMSD analysis revealed mean values of 0.273 ± 0.034 nm for 145398-61-4 and 0.255 ± 0.028 nm for L5F, with fluctuations generally ranging between 0.2 and 0.4 nm (Fig 3B).

Both the 145398-61-4 and L5F complexes displayed nearly identical fluctuation profiles, with peaks observed at corresponding regions of the protein (Fig 3C). The average backbone RMSF values were 0.060 ± 0.029 nm for 145398-61-4 and 0.059 ± 0.029 nm for L5F.

Both complexes, Keap1–145398-61-4 and Keap1–L5F, exhibited nearly identical Rg profiles throughout the simulation (Fig 3D). The mean Rg values were 1.792 ± 0.005 nm for 145398-61-4 and 1.791 ± 0.006 nm for L5F, with minimal fluctuations ranging from 1.77 to 1.81 nm.

**Fig 4:**
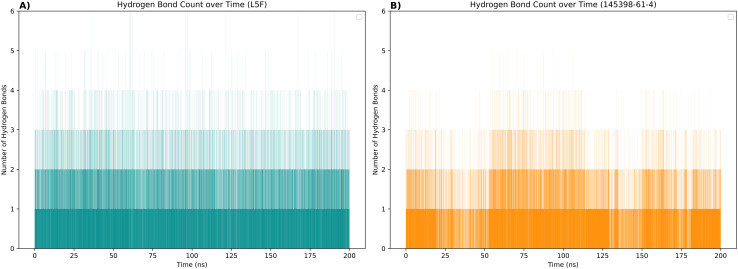
Hydrogen bond dynamics of Keap1–ligand complexes during molecular dynamics simulations. **(A)** The reference ligand L5F maintained a relatively stable hydrogen bond profile with Keap1, forming an average of 1–3 hydrogen bonds throughout the simulation period. (B) The ligand 145398-61-4 exhibited a higher frequency and number of hydrogen bonds compared to L5F.

Additionally, analysis of the simulation trajectories revealed that 145398-61-4 formed more polar interactions with Keap1 compared to the reference ligand L5F (Fig 4).

### Principal component analysis

**Fig 5:**
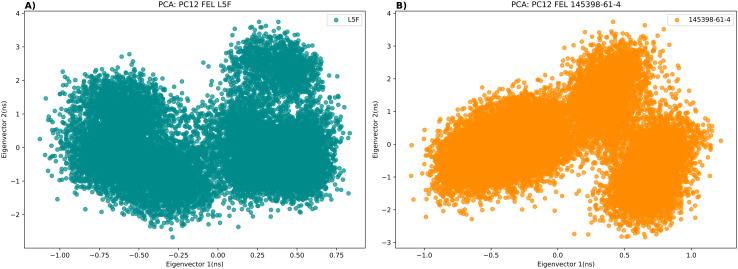
Principal component analysis (PCA) of the main motions in Keap1–ligand complexes during 200 ns molecular dynamics simulations. (A) Distribution of conformations for the L5F–Keap1 complex along Eigenvectors 1 and 2, showing a compact spread with two main clusters and one minor cluster. (B) Distribution of conformations for the 145398-61-4–Keap1 complex along Eigenvectors 1 and 2, showing a wider spread along Eigenvector 1 with distinct clustering patterns.

We used the PCA [54] to examine the main motions and dynamics of Keap1 when bound to L5F and 145398-61-4. The projections along the two principal components (Eigenvector 1 and Eigenvector 2) revealed slightly different but still stable conformational spaces for both complexes (Fig 5). The L5F-Keap1 complex displayed a more compact distribution with limited variation along both eigenvectors, indicating restricted flexibility and the presence of two main states and one minor state. Conversely, the 145398-61-4-Keap1 complex exhibited a broader spread along Eigenvector 1, but with distinct clustering patterns (Fig 5).

### Free energy calculation

The Molecular Mechanics-Poisson Boltzmann Surface Area (MM-PBSA) method is widely used in computer-aided drug discovery to estimate ligand-receptor binding free energies, effectively balancing computational speed and accuracy [55,56]. In this study, the binding free energy (ΔGbind) was calculated by summing the gas-phase interaction energy (ΔGgas) and the solvation energy (ΔGsol) as shown in Table 2. Among the energy components, van der Waals interactions contributed most significantly to the overall binding energy. The van der Waals energies were calculated as −44.88 kJ/mol for L5F and −42.50 kJ/mol for 145398-61-4, showing favorable non-bonded interactions for both ligands. The average total binding free energies were −32.40 kJ/mol for L5F and −27.12 kJ/mol for 145398-61-4.

**Table 2:** Binding free energies for the 145398-61-4-Keap1 and L5F-Keap1 complexes.

Metric	Ligands	VDWAALS	EEL	EGB	ESURF	GGAS	GSOLV	TOTAL
**Average**	145398-61-4	−42.50	−8.06	29.16	−5.71	−50.56	23.45	−27.12
	L5F	−44.88	−22.38	40.57	−5.71	−67.26	34.86	−32.40
**Std. Dev.**	145398-61-4	2.58	2.96	2.30	0.30	3.17	2.27	2.37
	L5F	1.93	1.46	1.37	0.12	2.67	1.38	3.00
**Std. Err. of Mean**	145398-61-4	0.12	0.13	0.10	0.01	0.14	0.10	0.08
	L5F	0.09	0.07	0.06	0.01	0.12	0.06	0.11

### HOMO-LUMO analysis

**Fig 6:**
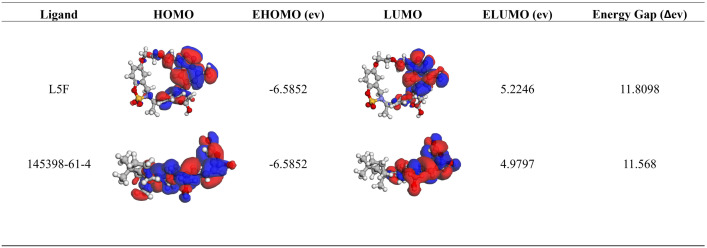
Analysis of HOMO and LUMO energies of L5F and 145398-61-4 ligands, along with their energy gaps (ΔE). Both L5F and 145398-61-4 show identical HOMO energy values (−6.5852 eV). However, the LUMO energy is slightly lower for 145398-61-4 (4.9797 eV) compared to L5F (5.2246 eV), leading to a marginally smaller energy gap (ΔE = 11.5648 eV vs. 11.8098 eV).

Molecular orbital energy calculations were performed to evaluate the electronic properties of the hit molecule compared to the reference ligand. The HOMO energy (EHOMO) was −6.5852 for both ligands, indicating similar electron-donating abilities (Fig 6). However, slight differences appeared in the LUMO energy (ELUMO), with L5F showing a value of 5.2246 eV and 145398-61-4 having a slightly lower value of 4.9797 eV. Consequently, the energy gap (ΔE), which reflects the molecules’ chemical stability and reactivity, was calculated to be 11.8098 eV for L5F and 11.5648 eV for 145398-61-4.

## Discussion

To the best of our knowledge, this study is the first to employ an integrated computational approach to identify potential marine-derived inhibitors of the Keap1-Nrf2 interaction, a key regulatory pathway in oxidative stress-related neurodegenerative disorders, such as AD. Current research efforts focus on identifying small molecules that can modulate the Keap1-Nrf2 interaction to enhance Nrf2 activity. In this context, the results of this study reveal 145398-61-4 as a promising ligand with a strong binding affinity and stability to Keap1, as demonstrated through virtual screening, molecular docking, protein–ligand interaction analysis, and molecular dynamics simulations.

The analysis of protein-ligand interactions revealed that 145398-61-4 interacts with the Keap1 Kelch domain, similar to that of L5F. It forms hydrogen bonds with the SER508 residue in the polar subpocket P1 [49] and hydrophobic interactions with Tyr525, which has been previously identified as one of the three binding “hot-spots” of Keap1 [50,57]. These traits are similar to those of known Keap1 inhibitors, such as KI696 and tiliroside, which have been shown in preclinical models to inhibit Keap1-Nrf2 PPI interactions and reduce oxidative stress [57,58]. These binding patterns indicate that 145398-61-4 may function via a comparable inhibitory mechanism, presenting potential therapeutic advantages.

MD simulations confirmed the stability of the Keap1–145398-61-4 complex, demonstrating continuous backbone stability, minimal RMSD fluctuations, similar RMSF and radius of gyration profiles, and well-defined conformational states throughout the simulation period. Hydrogen bond analysis revealed a narrower range of bonding fluctuations for 145398-61-4 compared to L5F, suggesting more stable interaction behavior. Principal component analysis confirmed that both complexes occupied similar conformational spaces, and free energy calculations indicated that 145398-61-4 has a binding affinity identical to L5F. Overall, these findings support the potential of 145398-61-4 as a stable Keap1 inhibitor.

HOMO–LUMO analysis also suggested that 145398-61-4 possesses favorable electronic properties and chemical stability, further supporting its potential for biological activity.

The biological importance of these results lies in the role of the Keap1–Nrf2 pathway in managing oxidative stress, which contributes to the development of neurodegenerative diseases such as Alzheimer’s, Parkinson’s, and Huntington’s disease [59]. Direct Keap1 inhibitors are more effective than indirect activators because they are more selective and less likely to cause adverse effects. Our findings suggest that 145398-61-4 may act as a direct Keap1 inhibitor, stabilizing Nrf2 and boosting the expression of antioxidant genes, which could provide benefits in treating neurodegenerative disorders [60,61].

In a 2017 study, Kerr et al. demonstrated that a new direct inhibitor of the Keap1-Nrf2 PPI protects primary mouse neuronal cultures from the synapto-toxicity of naturally formed Aβ oligomers [23], a pathological condition of AD that results in synaptic loss and reduced activity. Thus, 145398-61-4 is also predicted to show similar effects owing to its ability to disrupt the Keap1-Nrf2 PPI and ameliorate neurodegenerative conditions such as AD.

Preclinical and clinical studies conducted in the past ten years have shown the advantages of focusing on the Keap1-Nrf2 interaction in neurological disorders, and some inhibitors of this interaction are now in different phases of clinical trials [49]. While no FDA-approved Keap1-Nrf2 inhibitors exist, some strong and specific Keap1-Nrf2 interaction inhibitors are available for preclinical studies. To bridge this gap, it is conceivable that safer and more effective Keap1-Nrf2 inhibitors with unique chemotypes and advantageous pharmacokinetics would be required.

However, this study has some limitations. Computational predictions, while insightful, cannot fully capture the complexity of biological systems. Docking and MD simulations are affected by force field accuracy and simulation timescales, which may impact the precision of binding predictions. Biochemical binding assays and cellular experiments will be necessary to confirm the inhibitory action of 145398-61-4 and its potential as a treatment for neurodegenerative diseases. More evidence from wet lab experiments is needed to develop 145398-61-4 as a pharmacological inhibitor of the Keap1-Nrf2 protein-protein interaction and to advance the subsequent stages of drug development.

## Conclusion

Targeting oxidative stress by blocking the Nrf2-Keap1 interaction offers a promising approach for treating neurodegenerative diseases. Although significant preclinical and clinical work has been conducted, no FDA-approved drug that specifically disrupts this interaction is currently available. In this study, we employed a thorough in silico screening process on 14,492 compounds from the MNP library, resulting in the identification of compound 145398-61-4 as a potent and safe Keap1 inhibitor. This compound exhibited excellent pharmacokinetic properties, including oral bioavailability, the ability to cross the blood-brain barrier, and minimal toxicity. Molecular dynamics simulations confirmed stable binding between 145398-61-4 and Keap1, bolstering its potential as a lead candidate. However, future in vitro and in vivo experimental studies are required to validate these findings. Such efforts will aim to develop and optimize 145398-61-4 as a new inhibitor of the Keap1-Nrf2 interaction for treating neurodegenerative conditions like AD, multiple sclerosis, and amyotrophic lateral sclerosis.

## Supporting information

S1 File Marine natural products (MNP) library dataset. The MNP library was retrieved as an SDF file containing 14,492 small molecules, each identified by its individual CAS number.
(SDF)

S2 File Binding affinity results from AutoDock Vina. The binding affinity scores of 13,314 MNPs were obtained by performing molecular docking using AutoDock Vina. The compounds were ranked based on their binding scores, and only 1,329 ligands that had binding scores ranging from −10 to −13.7 were selected for ADMET prediction.
(XLSX)

S3 File Ro5-based druglikeness analysis of selected MNPs. Among 1,329 compounds, 252 were predicted to have druglikeness according to Lipinski’s Rule of Five (Ro5).
(XLSX)

S4 File In Silico toxicity profiling results. In silico toxicity profiling of the ligands was done using the Protox-3 web server and Osiris Property Explorer. Only 15 compounds were predicted to show no or mild toxicity in Osiris Property Explorer and belonged to toxicity class 6, as indicated by the Protox-3 web server.
(XLSX)
